# Impact of Tissue Thickness on Computational Quantification of Features in Whole Slide Images for Diagnostic Pathology

**DOI:** 10.1007/s12022-025-09855-2

**Published:** 2025-04-08

**Authors:** Manav Shah, António Polónia, Mónica Curado, João Vale, Andrew Janowczyk, Catarina Eloy

**Affiliations:** 1 Department of Biomedical Engineering, Emory University and Georgia Institute of Technology, Atlanta, GA USA; 2 Pathology Laboratory, Institute of Molecular Pathology and Immunology of University of Porto (IPATIMUP), Porto, Portugal; 3 School of Medicine and Biomedical Sciences, Fernando Pessoa University, Porto, Portugal; 4 Department of Oncology, Division of Precision Oncology, University Hospital of Geneva, Geneva, Switzerland; 5 Department of Diagnostics, Division of Clinical Pathology, University Hospital of Geneva, Geneva, Switzerland; 6 Pathology Department, Medical Faculty of University of Porto, Porto, Portugal

**Keywords:** Tissue section thickness, Digital pathology, H&E, Image-based biomarkers, Preanalytic variables, Quality control

## Abstract

Tissue section thickness (TST) is an understudied variable in digital pathology that significantly impacts both visual assessments and computational analyses. This study systematically examines the effects of TST on whole slide images (WSIs) and nuclear-level features using thyroid tissue samples (*n* = 144) prepared at thicknesses ranging from 0.5 to 10 µm. By minimizing preanalytical variables and batch effects, we aimed to isolate TST as the primary factor in our experiment. Visual assessments indicated that thinner Sects. (0.5–3 µm) were more transparent with distinct cellular features, while thicker Sects. (5–10 µm) appeared darker with increased staining intensity and artifacts. Quantitative analyses were performed using open-source tools such as HistoQC for WSI quality control, HoverFast for nuclear segmentation, and feature extraction with Scikit-learn and Mahotas. Both WSI and nuclear-level metrics were significantly influenced by TST. The Haralick texture feature of difference entropy, which measures texture complexity, showed a 13.7% decrease in nuclei as TST increased, indicating fewer complex textures in thicker sections. Additionally, intensity decreased substantially with thicker tissue, dropping by 26.1% at the WSI level and 30.4% at the nuclear level. WSI contrast exhibited an increase of 92.6% when transitioning from 0.5 to 10 µm. These findings demonstrate that variations in TST can obscure or alter the appearance of biological signals, complicating both visual diagnostics and computationally extracted features. The study highlights the need for standardized tissue section thickness protocols, alongside consistent reporting of these standards, to ensure accuracy and reliability in both visual evaluations and computational analyses within digital pathology workflows.

**Supplementary Information:**

The online version contains supplementary material available at 10.1007/s12022-025-09855-2.

## Introduction

In diagnostic pathology, the characterization of diseases typically requires processing tissue onto glass slides for microscopic observation. This includes steps such as fixation, tissue processing, embedding, sectioning, staining, coverslipping, and scanning. Variations in parameters used during these laboratorial steps have been shown to affect the presentation and quality of resulting slides [1–3].

**Fig. 1:**
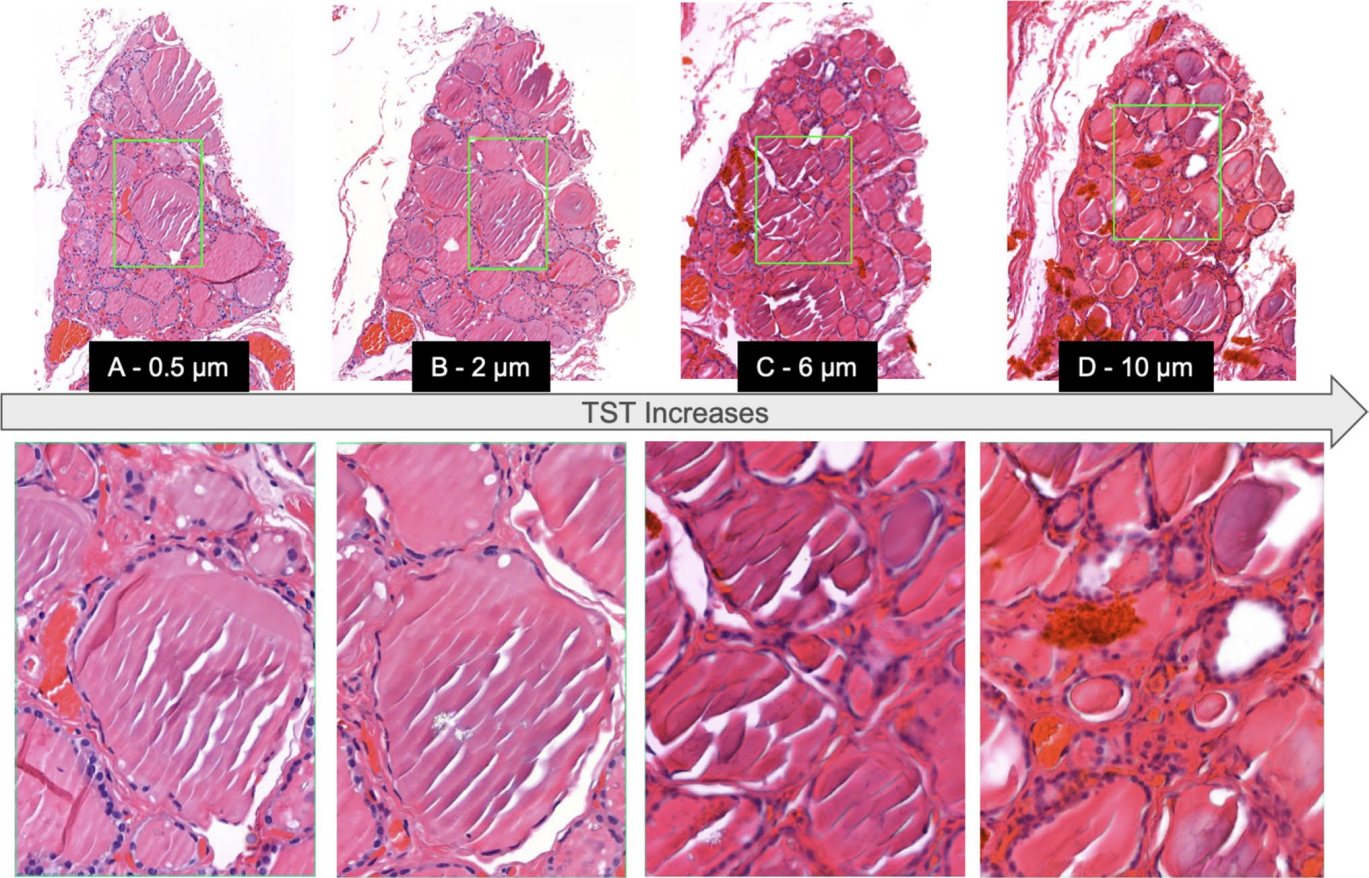
(Top) Images from consecutive cuts of tissue from the same tissue block at × 10. (Bottom) Associated × 20 magnification view of regions indicated by green box at × 10. It can be seen that as tissue section thickness (TST) increases, the tissue becomes darker and there is more blur artifact present. It is also apparent that in thicker sections, nuclear and structure boundaries are less well defined. It is evident that even sections taken from the same patient, at nearly the same location, can exhibit different characteristics attributable to TST variability.

One understudied parameter is tissue sectioning thickness (TST), which refers to the thickness of tissue slices cut during the sectioning process. While TST has been studied for some techniques, such as immunohistochemistry [4], the optimal thickness for producing hematoxylin–eosin (H&E) slides remains under-investigated. Diagnostic pathology laboratories individually establish TST values in their routine workflows, and thus, TST has been shown to range from 2 to 5 µm. TST-dependent variability is influenced by factors such as the skill of the histotechnician, the consistency of the tissue, and the quality of materials like the microtome blade. Additionally, differences in microtomes—including their calibration, maintenance, design, precision, and level of wear—further contribute to variations in section thickness and quality. TST and its internal variability have a clear impact on tissue presentation (see Fig. 1) which may potentially impact a patient’s diagnosis; however, this remains an open area for investigation.

The digital transformation of the pathology workflow has been instrumental for the integration of computational algorithms into pathology practice [5]. These computational algorithms, now often based on machine and deep learning techniques, have been shown to aid in the assessment of diagnostic, prognostic, and therapy predictive features [6–10]. Algorithms, serving as image-based biomarkers, are highly sensitive to tissue preparation steps that pathologists routinely account for, such as stain variability, site-specific batch effects (e.g., differences in tissue fixation, processing, and scanner calibration), and histological artifacts, all of which can compromise their performance [11–14]. Similarly, variable TST may introduce a non-biologically relevant signal, affecting visual and computational assessment of tissue features in WSI. Despite being a known visually assertable variable, TST and its impact on WSI presentation are yet to be studied.

This study explores the impact of TST on computational quantification of tissue characteristics, a crucial consideration as pathology increasingly relies on digital analysis. Thyroid tissue samples systematically prepared at various thicknesses to avoid batch effects were used to extract (a) slide-level features (e.g., brightness, contrast, homogeneity) and (b) nuclei-level features (e.g., morphological, intensity, and texture features). These features were then analyzed to determine how variations in TST influence these quantifiable metrics, revealing trends that may inform best practices in digital pathology.

## Materials and Experimental Design

### Sample Collection and Description

Between October 2023 to January 2024, 16 consecutive thyroid surgical specimens were received for observation at the Pathology Laboratory of IPATIMUP. For each specimen, a macroscopically normal tissue fragment without signs of diffuse disease was collected. The fragments measured 10 × 10 × 3 mm and had no representation of surgical margin. To avoid batch effects in data preparation, the following steps were taken: each of the 16 selected tissue fragments was processed (Donatello™ series 2 tissue processor, Diapath™, Martinengo, Italy) in a single cassette. After manual embedding, each of the 16 paraffin blocks was sectioned by the same histotechnician, using a single HistoCore BIOCUT® microtome (Leica Biosystems, Melbourne, UK), in two consecutive series of increasing thickness, i.e., {0.5, 1, 2, 3, 4, 5, 6, 10} µm. This process resulted in 16 slides for each patient, two at each thickness level. All slides were simultaneously stained in a single batch with H&E (Tissue-Tek Prisma® Plus automatic stainer, Sakura™, Tokyo, Japan) and coverslipped with film (Tissue-Tek Film®, Sakura™, Tokyo, Japan). All slides were then sequentially scanned on the Pannoramic 1000® Scanner (3DHISTECH Ltd, Budapest, Hungary) at × 40 (0.25 µm/pixel), using a × 20 objective magnification with a 2 × doubler, with the scanning protocol clinically validated for histological WSIs currently used in the laboratory and managed by SlideCenter Version 3.1 software (3DHISTECH Ldt, Budapest, Hungary) [15].

### Experimental Pipeline

**Fig. 2:**
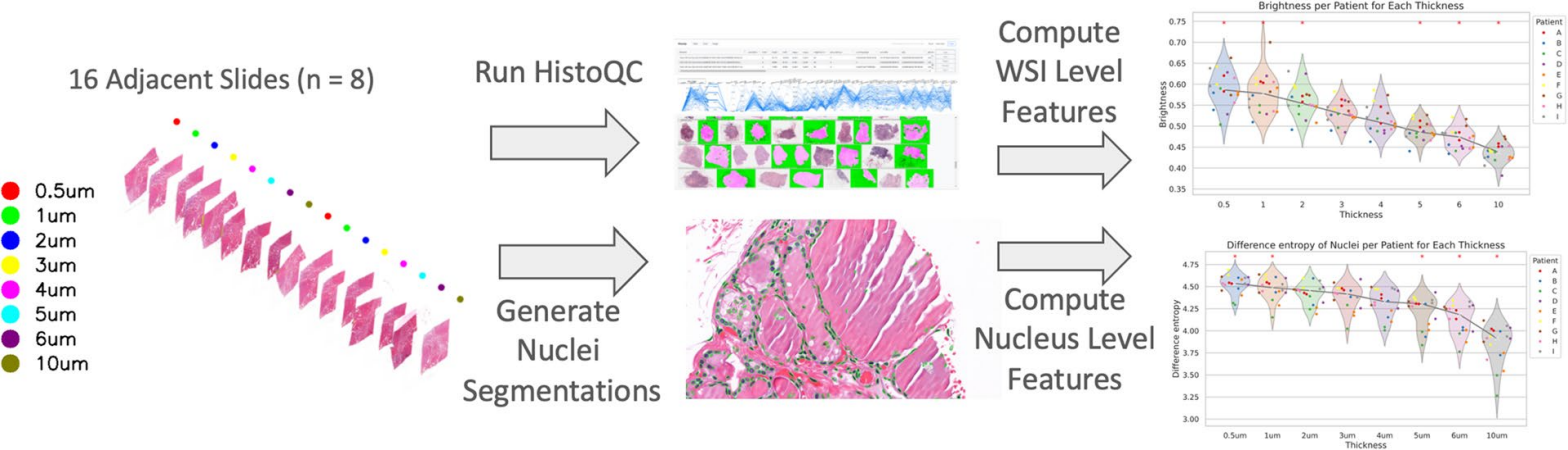
Workflow diagram illustrating feature extraction process from whole slide images: (left) Tissue blocks are sequentially sectioned twice at the same tissue thickness in a manner to limit inter-section batch effects; (middle) features are extracted at both (a) low magnification, using the open-source quality control tool HistoQC at the slide level, and (b) high magnification, focusing on nuclear features extracted with HoverFast, Scikit-learn, and Mahotas; (right) trends in resulting features are then analyzed as a function of tissue section thickness

Two experiments were performed at different levels of magnification (low vs high), via extracting (1) WSI-level and (2) nuclear-level features (see Fig. 2).

### Experiment 1: Evaluate Impact of TST on WSI-Level Features

**Fig. 3:**
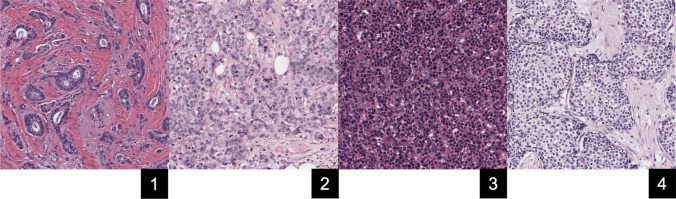
Reference images used by HistoQC for color distribution comparisons. Image 3 is analyzed in the manuscript and the only one that demonstrated obvious covariance with thickness. Mean squared error (MSE) was used to quantify differences between the color distributions of these reference images and analyzed images. Details of the MSE calculation can be found in the HistoQC documentation

WSIs were analyzed using HistoQC [16], a robust open-source quality control software designed for histopathology. A customized version of “config 2.1” was employed, which was tailored to exclude modules such as pen markings and crack detection—elements deemed irrelevant for this specific cohort based on visual assessment. For each WSI, HistoQC computed a comprehensive set of 90 quantitative metrics, which were stored in a tabular format. Additionally, the mean squared error (MSE) between a WSI’s color distribution and HistoQC-provided reference images was computed to quantify their relative differences. These images were chosen by the HistoQC team as images with acceptable but strikingly different H&E presentations (see Fig. 3). Features with strong covariance with TST, and relevance to image presentation, were chosen for more in-depth study (see Table 1).

**Table 1:** Whole slide image (WSI) features chosen for analysis with descriptions. The left column is the features selected. And the right column contains the descriptions of the features. These features capture visual properties of digital pathology slides, crucial for accurate analysis. Contrast highlights intensity differences, while brightness ensures clear visualization across slides. Mean squared error measures deviations in color from a reference. In concert, these features can help assess slide quality

Feature	Description
Contrast	A measure of the difference in intensity between adjacent pixels in a WSI and reveals insights into textures. Contrast is computed as a texture on the gray level co-occurrence matrix of 1000 32 × 32 patches from each slide (see Supplemental 4)
Brightness	The overall lightness or darkness of a digital pathology slide, important for visualizing tissue details and ensuring clear differentiation between the specimen and the background. The image is translated to grayscale, and the grayscale values are averaged over the whole image to calculate the grayscale brightness
Mean squared error	MSE compares the binned color distribution of all channels in an image to a reference image, providing a measure of similarity. This approach allows for the assessment of color consistency across tissue samples. This comparison helps assess deviations in color space

### Experiment 2: Evaluate Impact of TST on Nuclei-Level Features

Nuclei segmentation was performed using HoverFast [17], a high-throughput nuclei segmentation tool. This process yielded between 400,000 and 1,000,000 nuclei per slide, totaling 94,513,469 segmented nuclei. For each segmented nucleus, three families of features were extracted: (a) morphological features (e.g., area, eccentricity), (b) intensity features (e.g., mean, minimum, and maximum intensity), and (c) texture features (i.e., Haralick texture-based features [18]). These measurements were obtained using the Scikit-learn [19] and Mahotas [20] libraries, resulting in a feature vector length of 154 for each nucleus. Each feature value was averaged across all nuclei in the WSI. To ensure data quality, nuclei with intensity values below 10 and above 200, as well as area values below 50 pixels and above 1000 pixels at × 40 magnification, were excluded from the analysis. Visual confirmation of randomly selected regions from each thickness verified that most post-processed segmentations accurately represented nuclear regions. One feature from each feature family was selected for analysis (see Table 2).

**Table 2:** These nuclear features provide insights into cell morphology, intensity, and texture. The left column is the features selected, with the right column containing their description. Mean intensity reflects nuclear brightness, area measures nuclear size, and difference entropy captures texture complexity. Combined, they help characterize cellular structure in tissue samples

Feature	Description
Mean intensity	Intensity is the pixel value of the image when converted to grayscale. This is the same as brightness in WSI features. The average pixel value of all the pixels in a nucleus is taken
Area	The size of individual nuclei in the tissue sample
Difference entropy	The variation in intensity between neighboring pixels, representing the complexity of nuclear textures. This is one of many Haralick features [18] which are computed based on a gray co-occurrence matrix

Thyroid tissue was specifically selected for this study due to its homogeneity in cell type proportions, which enables meaningful cross-slide comparisons without the need to average over distinctly different cell populations. In our samples, the vast majority of cells are thyroid epithelial cells, providing a consistent and reliable basis for analysis. This experimental design was validated through visual confirmation, which showed minimal variation between slides in both cell types and their proportions. By leveraging this inherent consistency, we were able to confidently assess proportional distributions without requiring labor-intensive processes, such as individual nuclei labeling. This design choice ensures robust and efficient cross-slide comparisons.

### Statistical Analysis and Visual Assessment

**Fig. 4:**
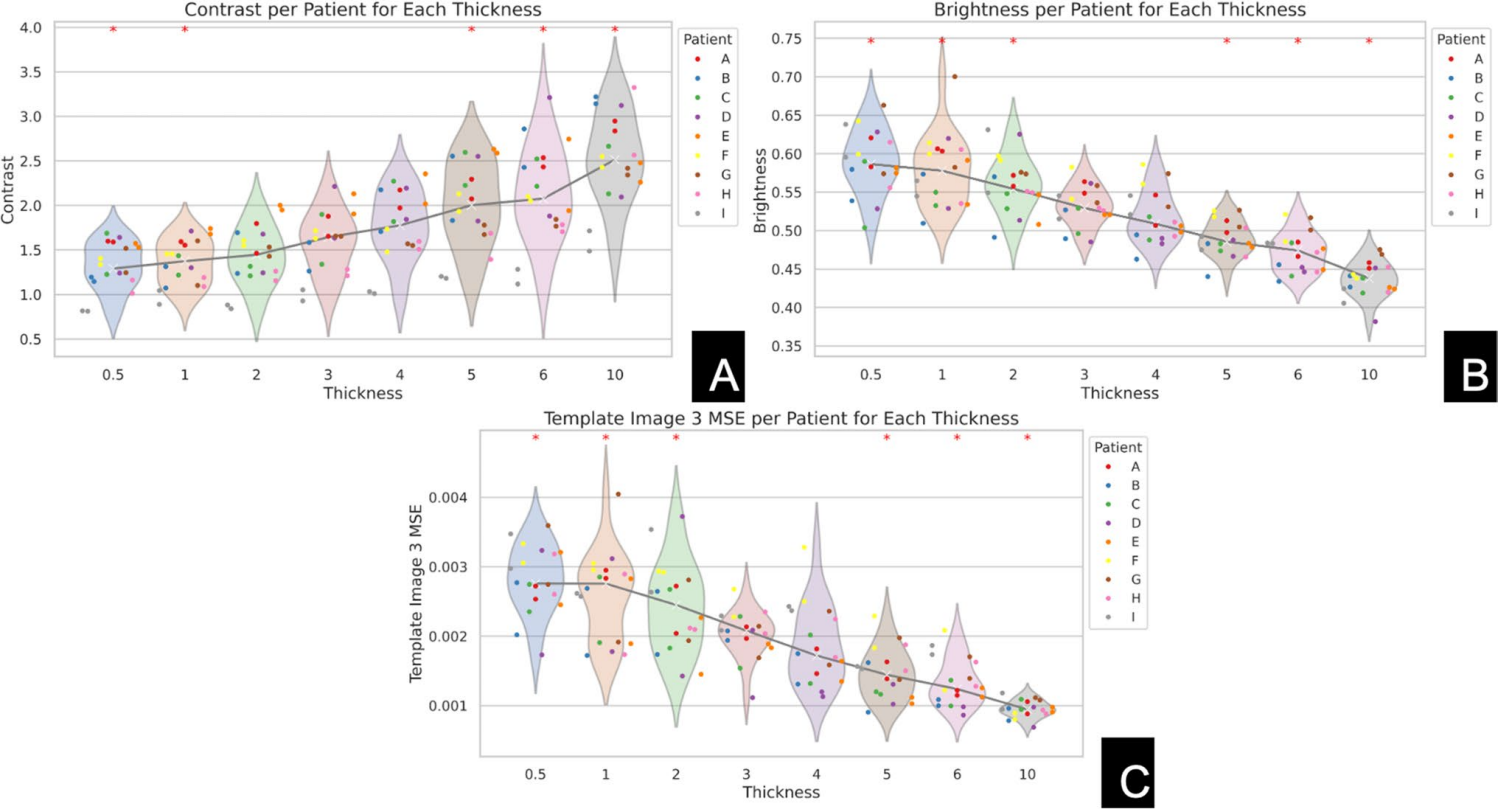
Violin plots depicting the relationship between tissue thickness and three image features: **A** contrast, **B** brightness, and **C** mean squared error (MSE) with reference image 3. The *x*-axis represents tissue thickness levels ranging from 0.5 to 10 µm, and the *y*-axis shows the corresponding values for each feature. Slides from the same patient are represented by dots of the same color. Statistically significant differences relative to the 3 µm thickness are indicated by an asterisk (*), based on pairwise *t*-tests. Thinner sections display increased brightness, while thicker sections exhibit higher contrast. MSE decreases as tissue thickness increases across several levels

Feature values were compared against the 3 µm as reference using pairwise *t*-tests with a significance level set at *p* = 0.01, before being visualized in violin plots (see Figs. 4 and 6). Three micrometer TST was selected as the reference point, as it represents a commonly used TST in clinical practice. Plots were reviewed to identify trends between feature value and TST. A thorough qualitative evaluation of the slides was conducted to observe the visual effects of tissue thickness on the presentation of both WSIs and nuclei. Lists of calculated features are found in the supplemental materials (see Supplemental 1, 2, 3).

## Results

### Feature Selection

After reviewing the violin plots to identify trends, the following features were selected for presentation and in-depth analysis: brightness, contrast, and MSE relative to a reference image to capture (WSI)-level characteristics (see Fig. 4). Nuclear features of area, difference entropy (a Haralick feature which quantifies complexity and randomness of texture patterns), and mean intensity were selected (see Fig. 6).

### WSI Features

#### Visual Assessment

Visual assessment of WSIs revealed distinct changes in tissue appearance with varying TST (see Fig. 1). Thinner Sects. (0.5 to 2 µm) exhibited greater transparency, with cellular and extracellular structures being more distinct and less obscured by overlapping tissue layers. As the tissue thickness increased (3 to 10 µm), the slides became progressively darker, with a noticeable reduction in the clarity of individual cells and structures. Denser tissue layers in thicker sections also introduced artifacts such as tearing, which were particularly prominent at 6 µm and 10 µm. The thicker sections appeared to contain a significant amount of blur imparted during the scanning process, likely due to TST’s impact on z-plane focusing algorithms [21].

#### Quantitative Assessment

The average brightness exhibited significant variation with tissue thickness. Thinner Sects. (0.5 to 2 µm) generally showed higher brightness levels compared to thicker Sects. (4 to 10 µm). Statistical tests (*t*-tests) confirmed that these differences were significant. Contrast also showed significant variation with tissue thickness. As the samples became thicker, contrast increased. Thicknesses of 0.5, 1, 5, 6, and 10 µm showed significance relative to 3 µm. Of the three reference images compared, only reference image 3 displayed a negative trend with thickness. As the thickness increased, the difference between the reference distribution of image 3 and the actual slide decreased, with thicknesses of 0.5, 1, 2, 5, 6, and 10 µm showing significance relative to 3 µm (see Fig. 3).

### Nuclear Features

#### Visual Assessment

**Fig. 5:**
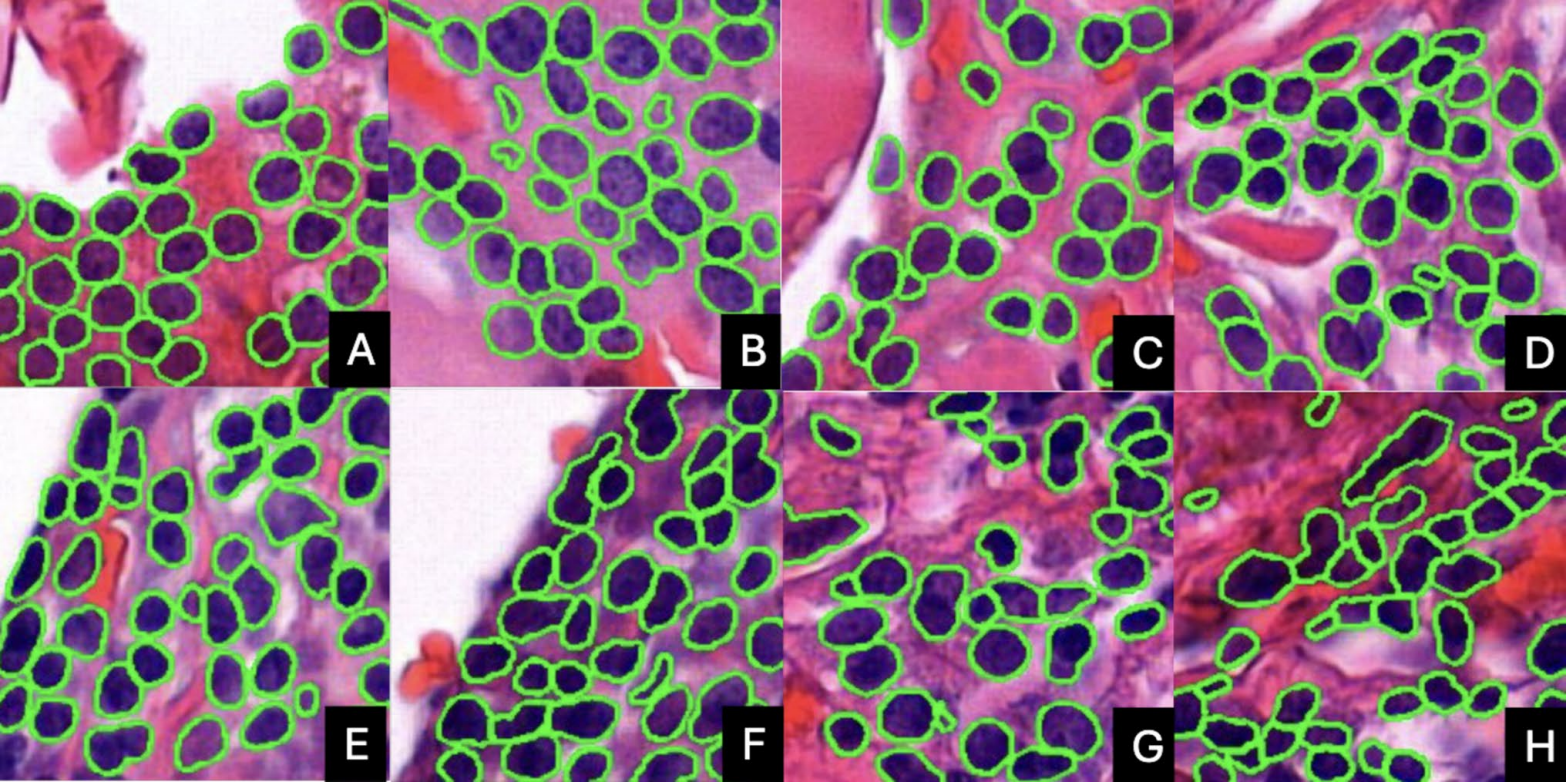
Samples of nuclear segmentations from each tissue thickness, illustrating variations in nuclear presentation as a function of tissue section thickness. The corresponding thickness levels are mapped as follows: **A** 0.5 µm, **B** 1 µm, **C** 2 µm, **D** 3 µm, **E** 4 µm, **F** 5 µm, **G** 6 µm, and **H** 10 µm. As tissue thickness increases, the nuclei become progressively darker. Additionally, nuclear boundaries appear less distinct, and textures become more homogenous at higher thickness levels, particularly in samples thicker than 4 µm

Nuclear presentation is noticeably affected by tissue thickness during visual assessment of histological sections. In thinner sections, such as those around 3 µm, nuclei appear lighter, and their boundaries are sharply defined, allowing for clear identification of individual nuclei. As tissue thickness increases, nuclei tend to appear darker. Further, in thicker sections, such as at 10 µm, multiple layers of nuclei can be visualized (see Fig. 5).

Nuclear segmentation quality showed some variation with tissue thickness. In thinner Sects. (0.5 to 3 µm), the nuclei were well delineated, allowing for accurate segmentation with high confidence. However, as tissue thickness increased, accuracy of segmentation was affected by factors like optical blur and artifacts, particularly in sections 5 µm and thicker. While this introduced some merging of adjacent nuclei and occasional misclassification of non-nuclear structures, the impact on overall segmentation remained within acceptable bounds for our measurements of texture and intensity features.

#### Quantitative Assessment

**Fig. 6:**
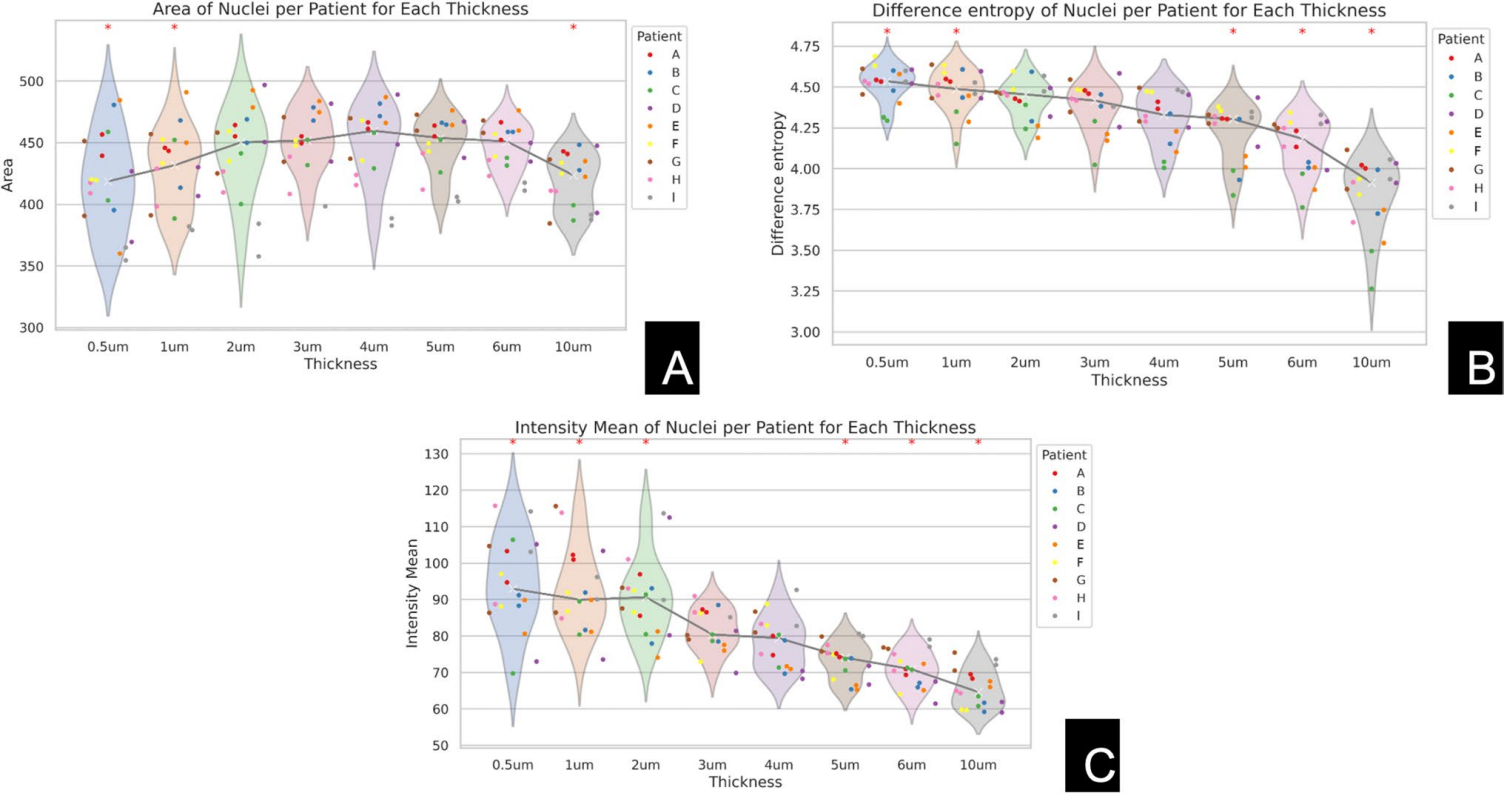
Violin plots depicting the relationship between tissue thickness and three image features: **A** area, **B** difference entropy, and **C** intensity. The *x*-axis shows tissue thickness levels (0.5 µm, 1 µm, 2 µm, 3 µm, 4 µm, 5 µm, 6 µm, 10 µm), and the *y*-axis represents the corresponding values for each feature. Slides from the same patient are represented by dots of the same color. For the area, tissue thicknesses up to 4 µm show an increase in area. For difference entropy and intensity, a general inverse trend is observed, with decreasing values as tissue thickness increases. Statistically significant differences relative to the 3 µm thickness are marked with an asterisk (*)

A noticeable increasing trend in nuclear area is observed as tissue thickness increases up to 4 µm (see Fig. 6a). This likely results from the higher probability of capturing the largest section of the nucleus in thicker cuts. The Haralick feature of difference entropy, which measures the complexity and randomness of texture patterns, showed significant variation with tissue thickness (see Fig. 6b). There was a downward trend with tissue thickness. Statistically significant differences were observed at thicknesses of 0.5, 1, 5, 6, and 10 µm compared to the 3 µm sections. There was also a noticeable decreasing trend in mean intensity as tissue thickness increased (see Fig. 6c). This decrease in mean intensity was statistically significant at thicknesses of 0.5, 1, 2, 5, 6, and 10 µm compared to the 3 µm sections.

## Discussion

This work studied how TST impacts the presentation of tissue, both visually and via quantitative characterization, at both slide and cell nuclear levels. Extensive laboratory efforts were made to eliminate sources of potential batch effects by processing all samples in a single batch for cutting, staining, coverslipping, and scanning. Slides were all prepared by the same technician in the same batch using the same equipment. Two slides from each patient were also taken to capture intra-patient variability and provide a more comprehensive context. To account for the possibility that adjacent slices from the same patient might display similar nuclear features due to shared structures, we ensured that slides taken at the same thickness were sampled from sections positioned as far apart as possible (see ordering in Fig. 2). In the end, in our well-controlled experiments, our results show significant covariation in tissue presentation with TST, suggesting the potential of TST as a confounding variable in downstream analysis.

TST plays a critical role in the current visual assessment of histology slides for diagnostic purposes. Optimal TST ensures cellular and structural details are clearly visible, allowing for accurate interpretation of tissue architecture and pathology. If TST is too thick, overlapping cells and structures can obscure critical diagnostic features, leading to potential misinterpretation or missed diagnoses. Conversely, overly thin sections may lack sufficient detail, making it difficult to identify key histological characteristics, such as nuclear morphology, mitotic figures, or subtle changes in tissue organization. Our assessment shows thinner sections (≤ 3 µm) appear to be the best for producing higher-quality slides, with our results demonstrating that thinner slides more clearly delineate cell and structure boundaries, while thicker sections begin to exhibit blurriness and overlapping structures.

Our results also strongly suggest that TST affects the visualization and quantification of features, from the overall color space to smaller nuclear details. As TST increases, the texture feature contrast rises due to differences between adjacent pixels, with the brightest regions often being the white background visible through tissue tears. Our study shows that increasing TST leads to a decrease in brightness, resulting in darker tissue overall. We hypothesize that this darker tissue, when set against the white background, enhances measured contrast. The finding that increased thickness predictably raises contrast is not immediately intuitive, as one might expect the darker tissue to diminish contrast in certain regions.

**Fig. 7:**
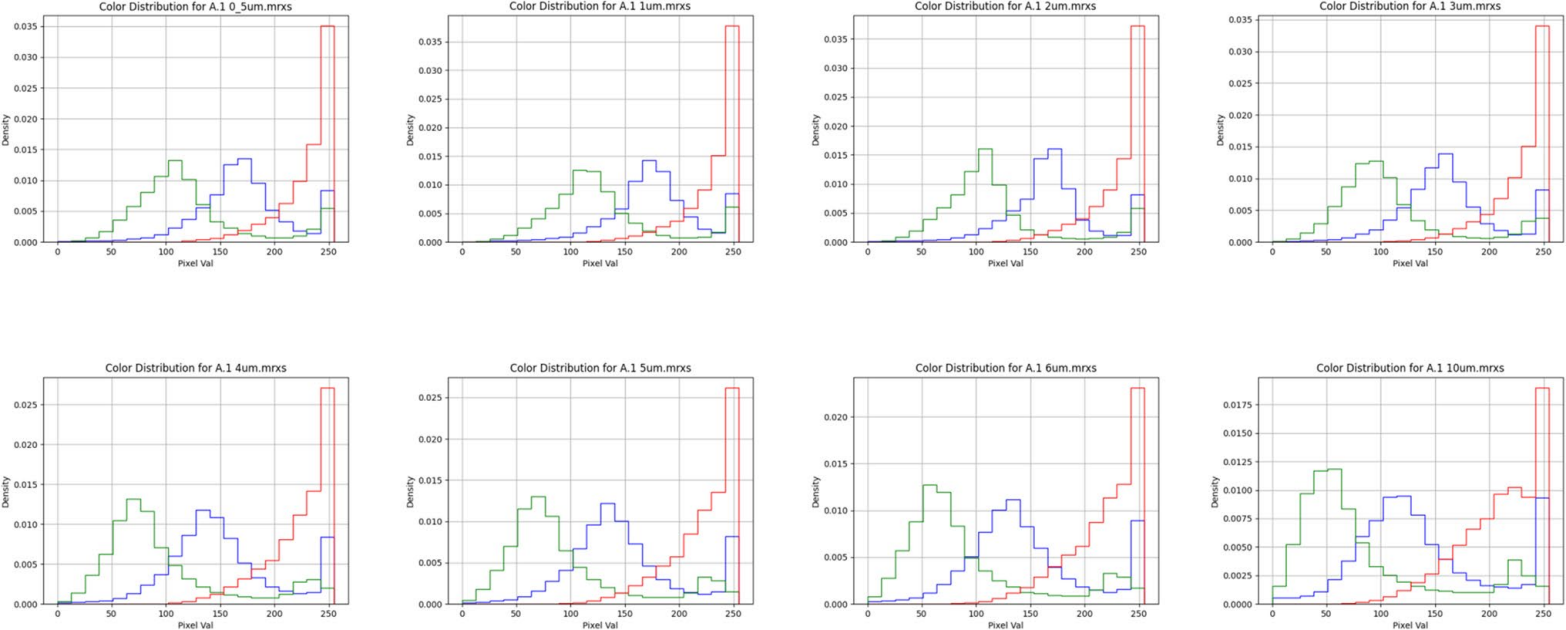
Color histograms from the same patient, produced by HistoQC, as TST increases from top-left (0.5 µm) to bottom-right (10 µm). While there is a noticeable leftward shift in the distributions toward lower intensities across all channels as TST increases, the blue and green channels appear to be most affected

In comparing the binned color distributions of the reference images to WSI using MSE, only one reference image, specifically the darkest one, exhibited a statistically significant trend. This result suggests that brightness, rather than broader variations in color across different channels, primarily influences the similarity between the color spaces of the images. Additionally, while all color channels exhibited a similar absolute drop in intensity, the green and blue channels were disproportionately affected in relative terms. Red channel brightness also minimally varied until 10 µm thickness (see Fig. 7 andSupplemental 5).

Intensity and brightness features have already been shown to vary with TST in studies in IHC [22]. Our study confirms these results in H&E on a WSI and nuclear level. This suggests that as TST increases, the amount of stain absorbed can change, affecting the intensity of the signal captured in the image. This, in concert with the Beer-Lambert law [23], in which the absorbance of light through a material is proportional to both the concentration of the absorbing substance and the path length (thickness), supports the notion that thicker tissues absorb more stain and more light.

When computed on segmented nuclei, the Haralick feature of difference entropy showed significant variation with TST. A potential explanation is that as more of the nucleus is included in thicker sections, the overlapping chromatin fibers within the nucleus interact with more stain creating a more homogenous texture. Increased homogeneity leads to lower difference entropy values. This effect appears to result in some level of covariation with all Haralick texture features with TST. Haralick features are commonly used in the development of image-based biomarkers [24]; thus, TST may negatively hamper their development, reproducibility, and reliability. Our results suggest that additional studies in this vein are warranted.

Another notable finding was the decreased clarity of nuclei boundaries in thicker samples (see Fig. 5), likely due to increased blurriness, which, despite good segmentation correspondence, may explain the collapse of the positive area trend at higher TSTs (see Fig. 6). Initially, we hypothesized that as thickness increases, the likelihood of capturing the widest part of the nucleus would increase, resulting in a larger average nuclear area. However, confirming this hypothesis is challenging due to the degraded clarity of the boundaries at higher thicknesses.

Z-plane focusing algorithms can account for some of these errors [25]. As TST increases, the potential for intra-slide variation in thickness may become more pronounced, causing the algorithm to focus sharply on structures within one plane while potentially blurring other structures located at different depths. This disproportionally affects thicker sections, where the increased depth and complexity make it difficult to consistently identify focal points which maintain clarity across the sample, leading to blur.

Although 10 µm samples are not common in clinical practice, they are increasingly required by spatial transcriptomics platforms to provide sufficient genetic material for the sequencing to succeed [26]. Researchers have suggested applying models trained on standard thicknesses to 10 µm spatial transcriptomics slides to correlate image-based features with genetic data [27]; however, our study indicates that this approach may be problematic, with models trained at thinner TSTs not being able to function optimally at higher TSTs.

Such disparities in tissue preparation highlight a key challenge in the reproducibility of machine learning models across diverse datasets, as site-specific variations have already been shown to introduce bias into deep learning algorithms [13]. To mitigate these effects, approaches like stain normalization [28] have been widely adopted. Our findings suggest that, in addition to these measures, sample TST should also be carefully considered as a factor influencing algorithmic performance. Appropriate laboratory quality control methods such as automatic sectioning machines and routine microtome calibration can help to ameliorate variable TST. However, inherent differences in, e.g., the fragments’ consistency, will remain key factors influencing TST irregularity. As such, while efforts to address TST variability are likely to yield improved consistency, it is unlikely that it can ever be entirely eliminated.

While directional trends can be observed—such as thinner tissue sections generally yielding higher-quality WSIs—defining specific optimal TST ranges for an algorithm remains challenging. This complexity arises from the interplay of multiple factors, including tissue characteristics, staining methodologies, and the specific algorithms employed for analysis. Algorithms trained on tissue from a particular TST range are likely to perform optimally within that range but may struggle with sections outside this learned distribution. As a result, acceptable TST ranges are often algorithm-specific rather than universally applicable. Additionally, different algorithms exhibit varying levels of robustness and failure characteristics. For example, low-magnification tasks, such as gross cancer detection, may remain relatively unaffected by TST variability due to the strength of the signal. In contrast, higher-magnification tasks that rely on more nuanced morphological features (e.g., mitosis detection) may be more sensitive to variations in TST, leading to increased susceptibility to failure. Unfortunately, many workflows currently do not mandate the documentation of TST, a gap that may hinder the reproducibility of studies and the reliability of diagnoses.

Addressing the lack of standardization requires a dual approach involving both clinical laboratories and developers of computational algorithms. From the clinical laboratory perspective, there is a need to harmonize and standardize tissue preparation practices, including the reporting of TST. On the computational side, algorithms should specify the TST they are optimized for and might also explore innovative solutions for automatically detecting and adjusting to it.

While our results are striking, there were however some limitations to this study. This included lack of a definitive ground truth for nuclei boundaries. There is a notable blur in the thicker sections, consequently affecting segmentation performance. Also, though HoverFast has demonstrated acceptable performance in the past, false positives are unavoidable when operating on such a large scale. To compensate, thorough data cleaning and visual verification were employed. This study also exclusively looks at thyroid tissue; however, it could be reasoned that similar trends will be present in a variety of tissue types. Also, this study exclusively analyzed benign tissue due to its consistent presentation, enabling a more controlled investigation of TST as the primary variable. In contrast, pathologic tissue can exhibit substantial heterogeneity due to the presence of disease, including variations in cellular architecture, stromal composition, and inflammatory response. These factors introduce additional complexity and could confound inter-patient TST comparisons. Given that we observed significant differences in TST even within the benign state, this highlights the need for follow-up studies that specifically examine TST in the context of distinct disease states.

Future research should aim to delineate how TST influences the diagnosis of malignancy and other pathological conditions while also focusing on developing standardized protocols for tissue sample preparation, establishing use case-specific thickness guidelines, and advancing computational algorithms that are more resilient to inherent tissue variability. This includes systematically testing algorithmic failure modes across different tasks and quantifying the impact of TST on existing biomarkers. A deeper understanding of these interactions will be crucial for ensuring the reliability and generalizability of machine and deep learning–driven pathology tools across diverse clinical settings. Lack of reproducibility in DP studies undermines the credibility of the field and hampers clinical implementation. By increasing the robustness of algorithms to pre-analytic variables, such as TST, the field of digital pathology can achieve more reliable and reproducible results.

## Conclusion

This study demonstrates that TST significantly affects both visual and computational analyses in digital pathology. Thinner sections generally show higher brightness and lower contrast, while thicker sections show decreased nuclear texture complexity and mean intensity. These variations can introduce noise into the biological signal that many image-based biomarkers are being trained to discover and employ for diagnosis, prognosis, and therapy response prediction. The standardization of tissue preparation may lead to improved diagnostic accuracy. Laboratories should document TST as part of the dataset metadata, enabling more consistent algorithm training and validation, and thus yielding more reliable applications in clinical settings.

## Supplementary Information

Below is the link to the electronic supplementary material.

ESM 1 (511 KB DOCX)

## Data Availability

Data generated or analyzed during this study are partially included in this published article and may be provided after reasonable request.
