# Correction: Impact of Tissue Thickness on Computational Quantification of Features in Whole Slide Images for Diagnostic Pathology

**DOI:** 10.1007/s12022-025-09872-1

**Published:** 2025-07-19

**Authors:** Manav Shah, António Polónia, Mónica Curado, João Vale, Andrew Janowczyk, Catarina Eloy

**Affiliations:** 1 Department of Biomedical Engineering, Emory University and Georgia Institute of Technology, Atlanta, GA USA; 2 Pathology Laboratory, Institute of Molecular Pathology and Immunology, University of Porto (IPATIMUP), Porto, Portugal; 3 School of Medicine and Biomedical Sciences, Fernando Pessoa University, Porto, Portugal; 4 Department of Oncology, Division of Precision Oncology, University Hospital of Geneva, Geneva, Switzerland; 5 Department of Diagnostics, Division of Clinical Pathology, University Hospital of Geneva, Geneva, Switzerland; 6 Pathology Department, Medical Faculty, University of Porto, Porto, Portugal

**Correction: Endocrine Pathology (2025) 36:10**

10.1007/s12022-025-09855-2

In the abstract of this article, the tool was incorrectly referenced as HoverNet when it should be HoverFast. The original article has been corrected.

