# Supplementary material for: Impact of Tissue Thickness on Computational Quantification of Features in Whole Slide Images for Diagnostic Pathology
Source: Endocr Pathol. 2025 Apr 8;36(1):10. doi: 10.1007/s12022-025-09855-2 (PMC11978545; doi:10.1007/s12022-025-09855-2)
Supplement: Supplementary file 1 — (511 KB DOCX) [file 12022_2025_9855_MOESM1_ESM.docx]

**Supplemental**

| **Morphological Features** | | | | | |
| --- | --- | --- | --- | --- | --- |
| area | area_bbox | area_convex | area_filled | axis_major_length | axis_minor_length |
| bbox-0 | bbox-1 | bbox-2 | bbox-3 | centroid_local-0 | centroid_local-1 |
| centroid_weighted_local-0 | centroid_weighted_local-1 | centroid_weighted-0 | centroid_weighted-1 | centroid-0 | centroid-1 |
| eccentricity | equivalent_diameter_area | euler_number | extent | feret_diameter_max | inertia_tensor_eigvals-1 |
| inertia_tensor-0-0 | inertia_tensor-0-1 | inertia_tensor-1-0 | inertia_tensor-1-1 | label | moments_central-0-0 |
| moments_central-0-1 | moments_central-0-2 | moments_central-0-3 | moments_central-1-0 | moments_central-1-1 | moments_central-1-2 |
| moments_central-1-3 | moments_central-2-0 | moments_central-2-1 | moments_central-2-2 | moments_central-2-3 | moments_central-3-0 |
| moments_central-3-1 | moments_central-3-2 | moments_central-3-3 | moments_hu-0 | moments_hu-1 | moments_hu-2 |
| moments_hu-3 | moments_hu-5 | moments_hu-6 | moments_normalized-0-2 | moments_normalized-0-3 | moments_normalized-1-2 |
| moments_normalized-1-3 | moments_normalized-2-0 | moments_normalized-2-1 | moments_normalized-2-2 | moments_normalized-2-3 | moments_normalized-3-0 |
| moments_normalized-3-1 | moments_weighted_central-0-0 | moments_weighted_central-0-1 | moments_weighted_central-0-2 | moments_weighted_central-0-3 | moments_weighted_central-1-0 |
| moments_weighted_central-1-1 | moments_weighted_central-1-2 | moments_weighted_central-1-3 | moments_weighted_central-2-0 | moments_weighted_central-2-1 | moments_weighted_central-2-2 |
| moments_weighted_central-2-3 | moments_weighted_central-3-0 | moments_weighted_central-3-1 | moments_weighted_central-3-2 | moments_weighted_central-3-3 | moments_weighted_hu-0 |
| moments_weighted_hu-1 | moments_weighted_hu-2 | moments_weighted_hu-3 | moments_weighted_hu-4 | moments_weighted_hu-5 | moments_weighted_hu-6 |
| moments_weighted_normalized-0-2 | moments_weighted_normalized-0-3 | moments_weighted_normalized-1-1 | moments_weighted_normalized-1-2 | moments_weighted_normalized-1-3 | moments_weighted_normalized-2-0 |
| moments_weighted_normalized-2-1 | moments_weighted_normalized-2-2 | moments_weighted_normalized-2-3 | moments_weighted_normalized-3-0 | moments_weighted_normalized-3-1 | moments_weighted_normalized-3-2 |
| moments_weighted-0-0 | moments_weighted-0-1 | moments_weighted-0-2 | moments_weighted-0-3 | moments_weighted-1-0 | moments_weighted-1-1 |
| moments_weighted-1-2 | moments_weighted-1-3 | moments_weighted-2-0 | moments_weighted-2-1 | moments_weighted-2-2 | moments_weighted-2-3 |
| moments_weighted-3-0 | moments_weighted-3-1 | moments_weighted-3-2 | moments_weighted-3-3 | moments-0-0 | moments-0-1 |
| moments-0-2 | moments-0-3 | moments-1-0 | moments-1-1 | moments-1-2 | moments-1-3 |
| moments-2-0 | moments-2-1 | moments-2-2 | moments-2-3 | moments-3-0 | moments-3-1 |
| moments-3-2 | moments-3-3 | orientation | perimeter | perimeter_crofton | quartiles-0 |
| quartiles-1 | quartiles-2 | shannon_entropy | solidity | x | y |

**Supplemental 1:** List of all Morphological features extracted. These are generated by the Scikit-learn library using regionprops.

| **Texture Features** |
| --- |
| Information Measure of Correlation 2 |
| Entropy |
| Sum Average |
| Contrast |
| Difference Variance |
| Sum of Squares: Variance |
| Sum Entropy |
| Angular Second Moment |
| Difference Entropy |
| Correlation |
| Information Measure of Correlation 1 |
| Sum Variance |
| Inverse Difference Moment |

**Supplemental 2**: List of all Texture features extracted. These were extracted using Mahotas which contains a Haralick feature extractor

| **Intensity Features** |
| --- |
| intensity_max |
| intensity_mean |
| intensity_min |

**Supplemental 3:** List of all Intensity features extracted. These are generated by the Scikit-learn library using regionprops.

$$\text{Contrast}=\sum_{i=0}^{N-1} \sum_{j=0}^{N-1} \left( i-j \right)^{2} P\left( i,j \right)$$

Where:

- $P\left( i,j \right)\text{ is the probability of occurrence of the pair of pixels with intensities }i\text{ and }j.$
- $N\text{ is the number of gray levels.}$

**Supplemental 4:** The formula for computation of contrast as computed by a gray level cooccurrence matrix (GLCM) on a patch extracted from the HistoQC computed tissue mask. The GLCM contrast was computed for each patch to quantify local intensity variation. In our study 8 gray levels were used as this is the default in HistoQC. Higher GLCM contrast values represent regions with greater intensity differences between neighboring pixels, indicating more pronounced texture, while lower contrast values suggest smoother areas. This metric was employed to assess the relationship between tissue thickness and texture features across varying regions of the slide, providing insights into morphological patterns associated with diagnostic variability.


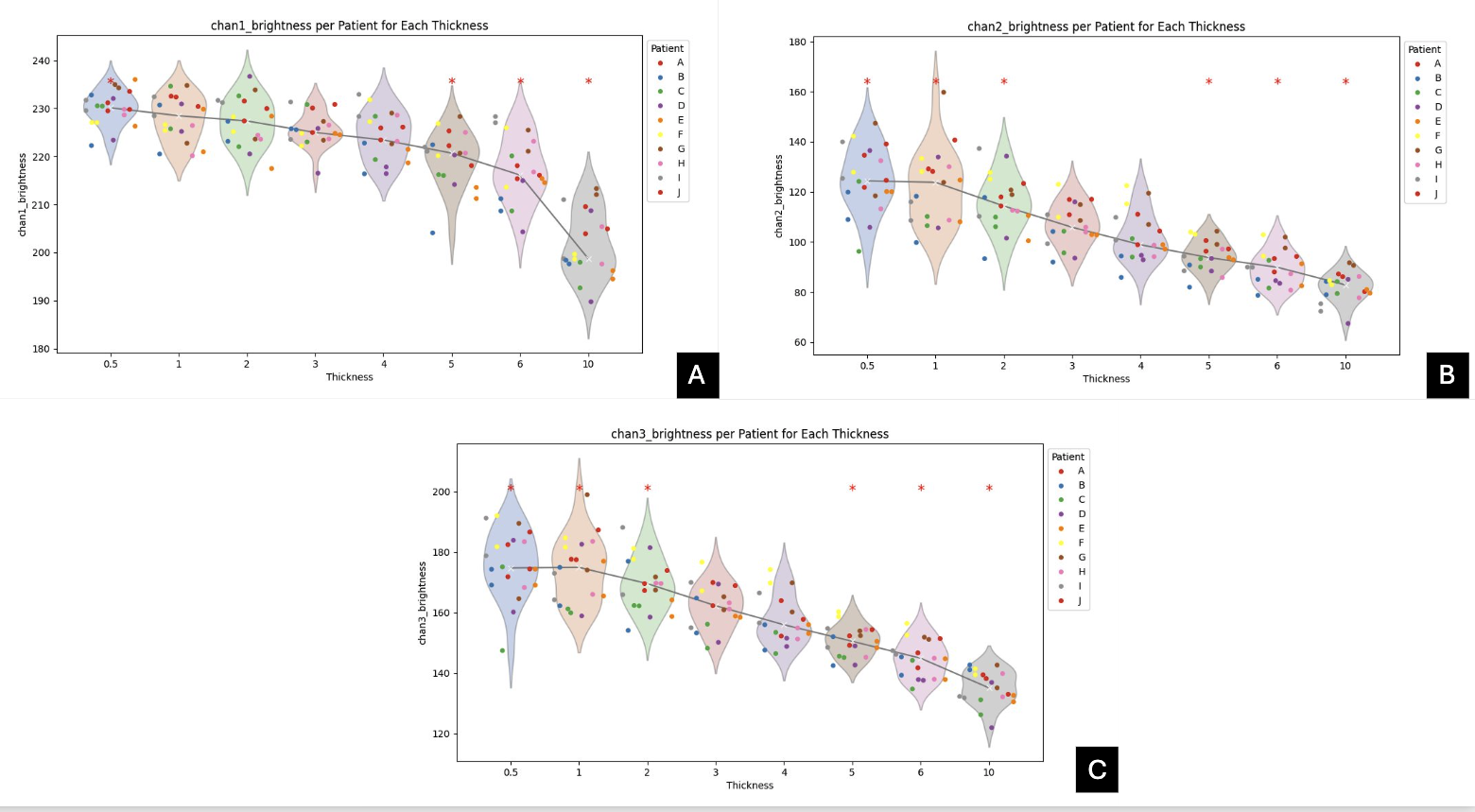


**Supplemental 5:** Violin plots depicting the relationship between tissue thickness and image channel brightness: (a) channel 1 brightness (red), (b) channel 2 brightness (green), and (c) channel 1 brightness (blue). The x-axis represents tissue section thickness (TST) levels ranging from 0.5 µm to 10 µm, and the y-axis shows the corresponding values for each feature. Slides from the same patient are represented by points of the same color. Statistically significant differences relative to the 3 µm thickness are indicated by an asterisk (*), based on pairwise t-tests. Across all channels, the average intensity decreases by approximately 40 points (on a 0-255 scale) as TST increases from 0.5 µm to 10 µm.
